# Artificial Intelligence-Based Decision Support System for UAV Control in a Simulated Environment

**DOI:** 10.3390/s26082436

**Published:** 2026-04-15

**Authors:** Przemysław Sujecki, Damian Frąszczak

**Affiliations:** Institute of Computer and Information Systems, Faculty of Cybernetics, Military University of Technology, 00-908 Warsaw, Poland

**Keywords:** UAV control, reinforcement learning, PPO, actor–critic, autonomous navigation, GPS-denied environment

## Abstract

Unmanned aerial vehicles (UAVs) are increasingly deployed in missions that require high autonomy and reliable decision-making; however, many operational concepts still assume access to GNSS and stable communication with a human operator. In contested environments, this assumption may no longer hold because GNSS degradation, radio-frequency interference, and intentional jamming can disrupt positioning and communication, thereby reducing mission effectiveness and safety. Recent surveys show that operation in GNSS-denied environments remains a major challenge and often requires alternative perception, localization, and control strategies. In response, this article investigates a reinforcement learning (RL)-based decision-support system for the autonomous control of a quadrotor UAV in a three-dimensional simulated environment. Rather than following pre-programmed waypoints, the UAV learns a control policy through interaction with the environment and reward-driven adaptation. The proposed system is designed for mission execution under uncertainty, limited external guidance, and partial observability. Two policy-gradient approaches are implemented and compared: classical REINFORCE and Proximal Policy Optimization (PPO) with an Actor–Critic architecture. The study presents the simulation environment, state and action representation, reward formulation, staged training procedure, and comparative evaluation. The results indicate that, within the considered unseen test scenario, the PPO-based configuration achieved higher mission effectiveness than REINFORCE in the final unseen test scenario, supporting the practical relevance of structured deep reinforcement learning for UAV operation in GPS-denied and communication-constrained environments.

## 1. Introduction and Research Motivation

Autonomous decision-making in complex and uncertain environments has become a key challenge in modern engineering systems. This is particularly evident in the domain of unmanned aerial vehicles (UAVs), which are increasingly expected to operate with minimal human supervision while performing tasks such as navigation, target reaching, and obstacle avoidance in dynamic three-dimensional environments. Traditional decision systems, typically based on predefined rules and control laws, become increasingly difficult to design, scale, and maintain as environmental complexity and the number of possible system states increase. While such rule-based approaches can be effective in well-defined and static scenarios, they struggle to adapt to uncertainty, nonlinearity, and unforeseen situations [1,2,3,4].

In contested and infrastructure-poor environments, autonomous UAV operation is further complicated by degradation or denial of external navigation and communication. Recent literature on GNSS-denied UAV navigation shows that reliable positioning and control remain difficult when satellite signals are obstructed, unavailable, or intentionally disrupted, and that robust operation increasingly depends on alternative sensing, sensor fusion, and adaptive decision mechanisms rather than on a single navigation source alone [5]. In parallel, security-oriented reviews on UAV navigation emphasize that spoofing and jamming of GNSS and communication links remain serious threats that may lead to loss of positioning, unstable control, or mission failure [6]. Therefore, the practical problem addressed in this paper is not jammer detection itself, but autonomous mission-level decision-making after external navigation and communication reliability has been degraded. Under such conditions, predefined control logic may prove insufficient because the UAV must react to incomplete observations, evolving threats, and degraded external support in real time. This motivates the use of adaptive learning-based decision mechanisms that can generalize beyond fixed rule sets and maintain mission effectiveness under operational uncertainty.

To address these limitations, learning-based decision systems have gained significant attention in recent years. Deep reinforcement learning (DRL) offers a principled framework for autonomous decision-making by enabling an agent to interact with its environment, learn control policies through reward feedback, and improve behavior without explicit programming of control rules [7]. The learned policy can be viewed as the agent’s “brain,” which makes decisions and is implemented as a deep neural network [8,9]. DRL has been applied to various UAV control tasks, including stable flight control, path planning, obstacle avoidance, and navigation in continuous action spaces [10,11,12]. For example, deep reinforcement learning based on Proximal Policy Optimization (PPO) has been explored to guide unmanned aircraft systems in continuous control environments, demonstrating high success rates in obstacle-avoidance tasks [13,14]. Furthermore, RL agents have been compared across multiple state-of-the-art algorithms, including PPO, Soft Actor–Critic (SAC), and Deep Deterministic Policy Gradient (DDPG), showing that policy-gradient and actor-critic methods often outperform traditional control techniques in uncertain environments [15,16].

Simulation environments and serious games play a crucial role in the development and evaluation of such learning-based decision systems [17,18]. They provide a safe, cost-effective, and fully controllable testbed in which complex scenarios can be reproduced without risking damage to real hardware. This is especially important for UAV systems, where real-world experimentation can be expensive, time-consuming, and potentially dangerous. Modern game engines, such as Unity, support the creation of realistic, physics-based environments and seamless integration with reinforcement learning frameworks via toolkits such as Unity ML-Agents, thereby enabling the study of continuous control policies in sophisticated 3D environments [19,20].

Although reinforcement learning has been widely studied for UAV control, the literature remains fragmented in its task definitions, learning setups, and evaluation metrics. Many studies focus on isolated subtasks such as attitude stabilization, obstacle avoidance, or path planning, often in simplified environments or with limited mission realism [2,13,21,22]. Recent reviews also note persistent gaps related to reward design, generalization, benchmark comparability, and deployment readiness, especially in GPS- or communication-denied settings [21,22]. At the same time, actor–critic methods, particularly PPO, are frequently favored in continuous-control settings because of their update stability and robustness. In contrast, simpler policy-gradient methods such as REINFORCE provide a useful baseline but often suffer from higher variance [2,13,21,22]. Consequently, there is still value in comparative studies that evaluate different policy-gradient strategies under the same sensing model, environment, mission constraints, and staged-training regime, because such a unified setup makes the effect of algorithmic differences easier to interpret.

This paper addresses the above gap by presenting an artificial intelligence-based decision system for autonomous control of a quadrotor UAV in a three-dimensional Unity/ML-Agents environment. The task under consideration is mission-oriented rather than narrowly low-level: the UAV must approach a designated target, avoid terrain obstacles, remain within operational boundaries, respect mission-time constraints, and maintain safe flight behavior. The proposed system is specifically motivated by GPS-denied and communication-constrained scenarios, in which the UAV should continue to act based on onboard observations and learned policy adaptation rather than on predefined trajectories or continuous operator guidance. Accordingly, the central research problem considered in this work is how to design and evaluate a UAV decision-making mechanism that remains effective when navigation support, communication reliability, and environmental predictability are all reduced. Two policy-gradient reinforcement learning configurations are implemented and compared: REINFORCE with a single policy network and PPO with separate actor and critic networks, clipped updates, and Generalized Advantage Estimation.

Two policy-gradient-based reinforcement learning configurations are implemented and evaluated: the REINFORCE algorithm using a single deep neural network, and the Proximal Policy Optimization (PPO) algorithm employing an Actor–Critic architecture with separate policy and value networks. The training process is conducted in multiple stages, with gradually increasing task complexity and a range of scenarios, enabling the emergence of increasingly sophisticated behaviors.

The main contributions of this work are as follows:

- Design and implementation of a realistic 3D UAV simulation environment for mission-oriented UAV control using Unity and ML-Agents, which is appropriate for reinforcement learning experiments requiring continuous control and physics-based interaction [8].
- Formulation of state representation, continuous action space, and reward function that jointly encode target approach, flight safety, obstacle avoidance, mission duration, and operational boundaries.
- Provide a controlled comparison between REINFORCE and PPO under the same sensing model, environment, mission constraints, and staged-training procedure, thereby isolating the effect of algorithmic differences under matched experimental conditions.
- Validate the final trained policies in a previously unseen scenario and show that, within the considered experimental setting, the PPO-based Actor–Critic configuration yields higher mission-level effectiveness than the REINFORCE baseline.
- Introduction of a staged training procedure in which task difficulty is increased progressively, and, at each stage, several candidate policies are trained under the current scenario setting. The best-performing policy from a given stage is then used to initialize training in the next, more demanding stage. This procedure was intended to improve learning stability and facilitate the gradual acquisition of mission-relevant behavior in a complex environment.

It should be emphasized that the contribution of this study is not the invention of a new reinforcement learning algorithm. Unlike many prior studies that focus on isolated subtasks such as obstacle avoidance, attitude stabilization, or waypoint following, the present work formulates a mission-oriented UAV control problem under degraded-navigation and communication-constrained conditions and evaluates two policy-gradient solutions within the same sensing, action, reward, and staged-training framework. The contribution, therefore, lies in the construction and experimental assessment of a coherent decision-support setup rather than in proposing a new RL optimizer.

The remainder of this paper is organized as follows. Section 2 presents the system concept and architecture, including the simulation environment, state and action representation, reward formulation, and reinforcement learning algorithms. Section 3 describes the staged training procedure and discusses the experimental results obtained for the REINFORCE and PPO configurations. Section 4 outlines directions for future work. Finally, Section 5 summarizes the main findings and conclusions of the study.

## 2. System Concept and Architecture

### 2.1. Problem Formulation

The decision system is responsible for controlling a virtual UAV (multirotor drone) in a bounded 3D environment. The main task of the UAV is to reach a designated target while:

1. Avoiding collisions with obstacles and the ground.
2. Remaining within a predefined operational area.
3. Completing the mission in a reasonable time.
4. Avoiding excessive maneuvering and unnecessary energy consumption.

**Figure 1:**
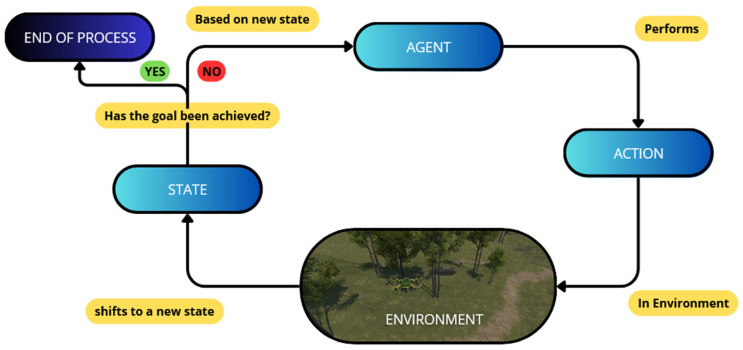
The reinforcement learning interaction loop is used in the proposed UAV control framework. At each time step, the agent receives an observation, selects a continuous action according to its policy, transitions to a new environment state, and receives a scalar reward reflecting mission progress, safety, and constraint satisfaction.

The UAV control task is formulated as a continuous-control Markov decision process [9] as presented in Figure 1. At each time step t, the environment occupies an underlying state $s_{t}\in S$, while the agent receives an observation $o_{t}\in O$, selects an action $a_{t}\in A$, transitions to a new state $s_{t+1}$, and receives a scalar reward $r_{t}\in R$. The policy is denoted by $\pi_{\theta }(a_{t}|o_{t})$, where θ  are the trainable parameters of the policy network. The objective of learning is to maximize the expected discounted return

$$G_{t}=\sum_{k=0}^{T-t-1}\gamma^{k}r_{t+k}$$

where γ∈(0,1] is the discount factor and T denotes the episode horizon.

In the present study, it is important to distinguish between the full environment state and the observation available to the agent. The state represents the complete simulator configuration, whereas the observation contains only the information accessible to the UAV through its onboard sensing and internal variables. This distinction is particularly relevant in GPS-denied settings, because the controller should act on partial and operationally plausible information rather than on privileged simulator state.

### 2.2. Modular Architecture

The system is designed in a modular fashion and comprises several cooperating components, each responsible for a distinct part of the training and evaluation process. At the core lies the simulation environment, implemented in Unity. Scripts that specify the physics of the UAV and its surroundings and control the flow of each training episode (initialization, progression, and termination conditions). Furthermore, the script prepares and sends observations and rewards to the reinforcement learning module and receives actions from it via the ML-Agents communication interface. The decision-making process is handled by the deep reinforcement learning module, implemented in Python version 3.10.0 using PyTorch version 2.3.1 + cuda 11.8 and the ML-Agents Python API version 0.30.0. This module performs policy inference, i.e., selects an action based on the current observations received from Unity with a stochastic type:

$$\pi \left(a_{t}|s_{t}\right)=P\left[A|s_{t}\right]$$

where

- $a_{t}$—Action, $a_{t}\in \;R^{k},$ where k is the number of dimensions of the action space. $R^{k}$ denotes the space of real-valued vectors used to describe the possible actions.
- $s_{t}$—State, $s_{t}\in$ $R^{w}$, where w is the number of dimensions of the state space. $R^{w}\;$ denotes the space of real-valued vectors used to describe the simulator state.
- A—The set of all possible, admissible actions. For discrete actions, A={0,1,2,3,…, n−1}, where n is the number of possible actions. For continuous actions $A\in R^{n}$, where n is the dimension of the action space. $R^{n}$ denotes the n-dimensional space of real-valued vectors, meaning that each action is represented by n  continuous numerical values.
- $\pi \left(a_{t}|s_{t}\;\right)$—The policy, which defines the probability distribution over actions $a_{t}$ in state $s_{t}$.
- $P\left[A|s_{t}\right]$—The probability of selecting a particular action $a_{t}$ from the set of all available actions A in given state $s_{t}$.

During training, it stores entire trajectories of interaction and computes returns and advantages for each time step:

$$R\left(\tau \right)=\;\sum_{k=0}^{\infty }\gamma^{k}r_{t+k+1}$$

where

- τ—Trajectory, a sequence of state–action pairs from time step t to ∞. It captures the agent’s entire trajectory.
- k—Index of successive time steps.
- γ—Discount factor for future rewards, γ∈[0,1];
- $r_{t}$—Reward obtained after performing a given action in a given state $r_{t}\in R$.

Based on these quantities, the learning module updates the parameters of the neural network according to the selected algorithm—REINFORCE or Proximal Policy Optimization (PPO).

To support the training process, monitoring and analysis tools are used, with TensorBoard as the primary tool. These visualizations enable the assessment of convergence, the detection of instabilities, and the systematic comparison of model variants, such as alternative network architectures or hyperparameter settings.

This modular design facilitates future changes to individual components (e.g., replacing the learning algorithm, modifying the environment, or changing the observation space) without restructuring the entire system.

### 2.3. Simulation Environment

**Figure 2:**
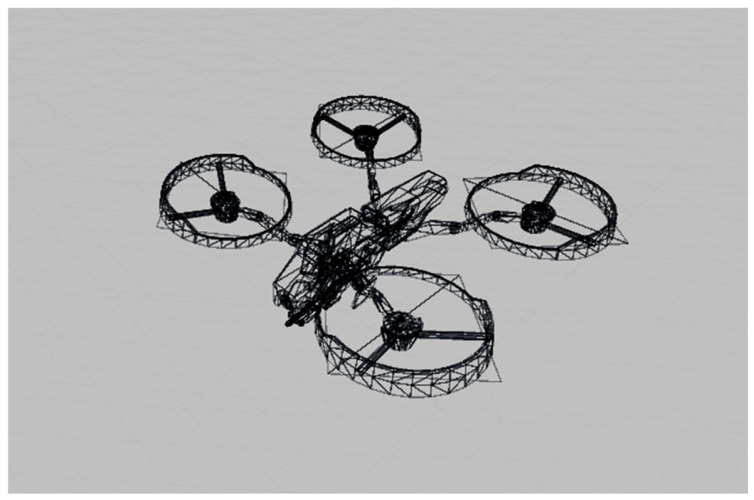
3D model of UAV.

The 3D simulation environment includes the target marker, the ground surface, and trees representing ground obstacles. The multirotor UAV model operating within the bounded mission area is shown in Figure 2.

The drone model was designed to capture the main qualitative components of multirotor flight dynamics, including rotor thrust, gravity, aerodynamic drag, and rotational torques, as well as cascaded PID stabilization. The simulator is therefore intended as a physics-informed training environment rather than as a high-fidelity aerodynamic replica of a specific real UAV platform. A cascaded PID control system (attitude and position loops) is used and carefully tuned to balance responsiveness and stability, so that the UAV’s simulated motion closely resembles real-world behavior and is suitable for testing control and navigation algorithms [23].

Three training configurations are defined, with increasing difficulty:

- a simple scenario without obstacles, used for initial training and stabilization;
- scenarios with one or more obstacles placed along the path to the target;
- variations in the target location and obstacle layout.

The scenarios were deliberately ordered from simplest to increasingly complex. The purpose of this design was to introduce the model to new skills gradually, rather than “throwing it in at the deep end.” Analogous to a child who does not start learning to ride a motorcycle but instead learns on a bicycle with training wheels, the model first learns basic behaviors in a simple environment and only later tackles more complex tasks. Additionally, a separate test scenario is created that differs from the training configurations in terms of the obstacle layout and target position. This enables assessment of the trained agent’s generalization capabilities [24].

### 2.4. State and Action Representation—Dataset

In this work, the dataset is explicitly defined by the authors. Here, the dataset consists of observations, i.e., data collected from sensors specified in the simulation. The number and configuration of these sensors are chosen by the author, and they collect data such as distance to the ground, obstacle detection when an obstacle is detected, and target detection when the target enters the sensor’s range. These perception sensors are Ray Perception Sensors. In addition to these external sensor readings, internal UAV-system observations are included, bringing the total number of observations to 146. All these observations constitute the input data for each simulation step of the artificial neural network model. Furthermore, because the approach is based on reinforcement learning, rewards and actions are integral to the data used to train the agent. The intent was to provide the policy with sufficient information for collision-aware navigation and target-oriented control without exposing privileged simulator-state variables unavailable to an onboard autonomous system. The observation design was determined empirically during simulator development: a smaller sensor set led to insufficient environmental coverage, whereas a denser set increased input dimensionality without a clearly proportional practical benefit. Although the final observation vector was found to be operationally effective, the present study does not yet include a formal feature-ablation or sensitivity analysis. Therefore, the 146-dimensional representation should be interpreted as an engineering design choice for this environment rather than as a proven minimal or optimal state representation. The purpose of the present work was to evaluate the overall decision-making framework and compare two reinforcement-learning strategies under a fixed sensing configuration, rather than to optimize the observation space itself. For this reason, the current manuscript does not claim that the adopted 146-dimensional observation vector is minimal, unique, or universally optimal.

**Figure 3:**
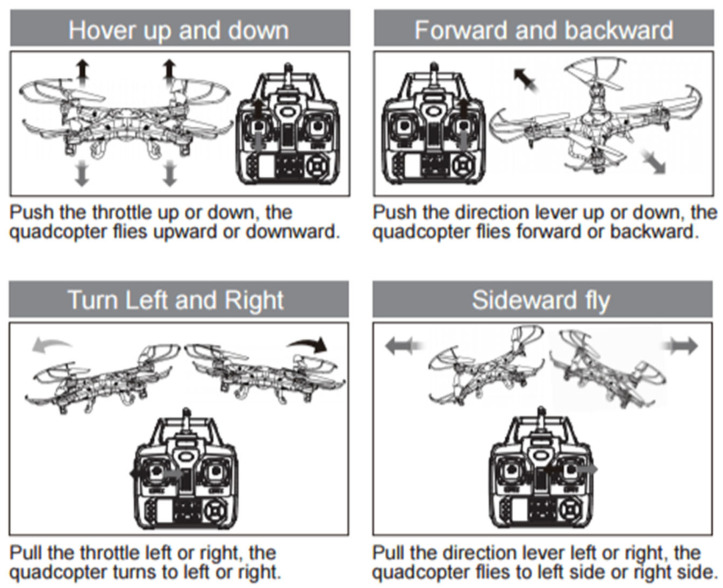
Available actions to perform by the AI Agent. Available online: https://www.habrador.com/tutorials/pid-controller/3-stabilize-quadcopter/ (accessed on 8 February 2025).

The action space is continuous. An action consists of control values that are mapped to the UAV’s equivalent low-level control inputs (e.g., thrust and attitude commands) as seen in Figure 3. Continuous actions enable smooth control and are well-suited to policy-gradient algorithms.

The processed observations are fed into a neural network policy, which outputs the corresponding continuous control actions.

### 2.5. Reward Shaping

The reward function is a key component of the system, as it defines the learning objective. The main reward components used in the proposed setup are summarized in Table 1. In the presented work, the reward at each step is constructed from several terms:

- Penalty for rapid throttle reversals;
- Reward for maintaining good height alignment with the target;
- Penalty for being far from the target’s height;
- Penalty for throttle usage;
- Penalty for excessive accumulated rotation;
- Reward for moving closer to the target;
- Penalty for moving away from the target;
- Penalty for falling below a minimum height;
- Penalty for leaving the allowed radius;
- Penalty for exceeding the maximum number of time steps;
- Large reward for reaching the target;
- Penalty for hitting an obstacle (trigger event);
- Penalty for hitting the ground (collision);
- Penalty for hitting an obstacle (collision);

The coefficients of the individual terms are tuned experimentally. The aim is to provide sufficient guidance so that the agent can learn meaningful behavior (e.g., moving toward the target, avoiding obstacles) without over-constraining the policy [25].

**Table 1:** Main reward components used in the proposed reward-shaping function, including their purpose, activation condition, and qualitative effect of weight magnitude on agent behavior.

**Training Objective/Reward Component**	**Description**	**Type**	Trigger/Activation Condition	Effect of Weight Magnitude
Approach Target	The agent receives a positive reward for each time step in which it reduces the distance to the designated target point. This encourages continuous progress toward the target.	Positive	Activated when $d_{t}<d_{t-1}$	Higher values strengthen direct target-seeking but may override safety or stability; lower values may weaken progress toward the target.
Deviation from Target	A penalty is applied when the UAV increases its distance from the target, discouraging inefficient or incorrect maneuvers.	Negative	Activated when $d_{t}>d_{t-1}$	Higher values more strongly suppress movement away from the target but may reduce maneuver flexibility; lower values may insufficiently discourage detours.
Mission Complete Time	A positive reward proportional to the speed of mission completion—faster target reaching leads to higher reward values.	Positive	Activated only at successful mission termination	Higher values promote faster mission completion but may encourage overly aggressive behavior; lower values may not sufficiently reward efficiency.
Operational Zone Compliance	The agent is penalized for leaving the predefined mission area or crossing operational boundaries.	Negative	Activated when the agent exits the allowed operational area	Higher values enforce boundary adherence more strongly but may make the agent too conservative; lower values may not sufficiently prevent zone violations.
Flight Efficiency	Penalty for unnecessary, excessive, or oscillatory maneuvers (e.g., frequent sharp turns or altitude changes without tactical need).	Negative	Activated when motion smoothness or control-effort thresholds are exceeded	Higher values reduce unnecessary maneuvers but may suppress needed corrections; lower values may not sufficiently limit wasteful motion.
Stable Flight Behavior	Additional reward for maintaining a smooth trajectory, minimizing abrupt attitude or velocity changes, and conserving energy.	Positive	Activated continuously when stability-related criteria are satisfied	Higher values promote smoother flight but may weaken task-oriented decisiveness; lower values may not sufficiently support stable behavior.

### 2.6. Learning Algorithms

Two policy gradient reinforcement learning algorithms are implemented and compared in the system:

1. REINFORCE—Classic Monte Carlo policy-gradient algorithm, which means that the agent collects complete episodes, computes the return (cumulative discounted reward) for each time step, and updates the policy parameters in the direction of the gradient of the expected return. Although conceptually simple, REINFORCE can exhibit high variance and slow convergence, particularly in complex environments [26,27]. The policy is represented by a deep neural network that outputs a probability distribution over actions, making the method inherently stochastic. Exploration is therefore constant in time and depends only on the spread of this distribution around its mean. To improve learning efficiency, dynamic exploration is additionally introduced. The REINFORCE objective is:

$$L^{PG}\left(\theta \right)=E_{t}\left[log\;\pi \left(a_{t}|s_{t}\right)\times R_{t}\right]$$

where

- $E_{t}$—Expected value at a given time step, $E_{t}$.
- θ—policy parameters $\pi \left(a_{t}|s_{t}\right)$—trainable parameters of the policy network.
- $a_{t}$—Action, $a\in \;R^{k}$, where k is the number of dimensions of the action space or $a_{t}\in \{0,1,2,\ldots ,n-1\}$, where n is the number of possible, allowed actions.
- $s_{t}$—State, $s_{t}\in$ $R^{w}$, where w is the number of state dimensions $s_{t}\in \{s_{1},s_{1},\ldots \;s_{n}\}$, where n is the number of possible, allowed states.
- $log\;\pi \left(a_{t}|s_{t}\right)$—logarithm of the probability of taking a specific action in this state, $log\;\pi \left(a_{t}|s_{t}\right)\in R$.
- $R_{t}$—Cumulative reward collected from the beginning of the episode until its end, $R_{t}\in R$.

2. Proximal Policy Optimization (PPO)—Actor–Critic method that combines policy and value function with Temporal-Difference (TD) Learning. In TD learning, value estimates are updated via bootstrapping: the current estimate is adjusted using the observed reward and the estimated value of the next state, without waiting for the episode to complete. In the traditional approach to updating the model (policy gradient), the objective function is defined as:

$$L^{PG}\left(\theta \right)=E_{t}\left[log\;\pi \left(a_{t}|s_{t}\right)\times A_{t}\right]$$

where

- $E_{t}$—Expected value at a given time step, $E_{t}$.
- θ—policy parameters $\pi \left(a_{t}|s_{t}\right)$ things like weights, biases, hyperparameters in the case of creating it using neural networks, or otherwise the parameters of a mathematical function. $\theta \in R^{d}$, where d—number of parameters.
- $a_{t}$—Action, $a\in \;R^{k}$, where k is the number of dimensions of the action space or $a_{t}\in \{0,1,2,\ldots ,n-1\}$, where n is the number of possible, allowed actions.
- $s_{t}$—State, $s_{t}\in$ $R^{w}$, where w is the number of state dimensions $s_{t}\in \{s_{1},s_{1},\ldots \;s_{n}\}$, where n is the number of possible, allowed states.
- $log\;\pi \left(a_{t}|s_{t}\right)$—logarithm of the probability of taking a specific action in this state, $log\;\pi \left(a_{t}|s_{t}\right)\in R$.
- $A_{t}$—scalar result of the advantage function, $A_{t}\in R$.

PPO introduces an important mechanism—clipping to prevent destructive policy updates. The algorithm employs a clipped objective function to constrain the magnitude of policy updates, thereby improving training stability [28]:

$$L^{CLIP}\left(\theta \right)=E_{t}\left[min(r_{t}\left(\theta \right)A_{t},\;clip\left(r_{t}\left(\theta \right),1-\epsilon ,1+\epsilon \right)A_{t})\right]$$

where

- $E_{t}$—Expected value at time a given time step, $E_{t}\in R$.
- $r_{t}\left(\theta \right)$—the ratio of the new policy to the old policy:
  $$r_{t}\left(\theta \right)=\frac{\pi_{\theta }\left(a_{t}|s_{t}\right)}{{\pi_{\theta }}_{old}\left(a_{t}|s_{t}\right)}$$

If $r_{t}\left(\theta \right)$ > 1, then the action is more likely to be taken under this policy than under the old one.

If 0 < $r_{t}\left(\theta \right)$ < 1, then the action is less likely to be taken under this policy than under the old one.

- $r_{t}\left(\theta \right)A_{t}$—The part that’s being clipped
- $clip\left(r_{t}\left(\theta \right),1-\epsilon ,1+\epsilon \right)$—A clipping function that clips $r_{t}\left(\theta \right)$ in range [1−ϵ,1+ϵ]
- $A_{t}$—scalar advantage value, $A_{t}\in R$.

In this work, Generalized Advantage Estimation is employed to compute advantage values [26,27]. GAE (Generalized Advantage Estimation) is a method used in policy-gradient reinforcement learning to compute a smoother, less noisy estimate of the advantage function.

Instead of using a single-step or fixed-k-step advantage, GAE combines multiple n-step advantage estimates with exponentially decaying weights [16]. This gives a compromise between:

- low variance (more stable learning);
- low bias (more accurate estimate of the true advantage);

GAE is controlled by a parameter λ (lambda). By tuning λ, one can adjust the relative weight of longer-horizon versus more immediate information. The generalized advantage estimate is defined as:

$${A\left(t\right)}^{GAE(\gamma ,\lambda )}=\;\sum_{l=0}^{\infty }{\left(\gamma \lambda \right)}^{l}\delta_{t+l}$$

where

- λ—GAE parameter, which can be tuned to trade off variance and bias λ∈[0,1];
- γ—The discount factor for future rewards, γ∈[0,1];
- l—Index of successive time steps;
- $\delta_{t}$—The temporal-difference error, $\delta_{t}\in R$.

### 2.7. Neural Network Architecture

**Figure 4:**
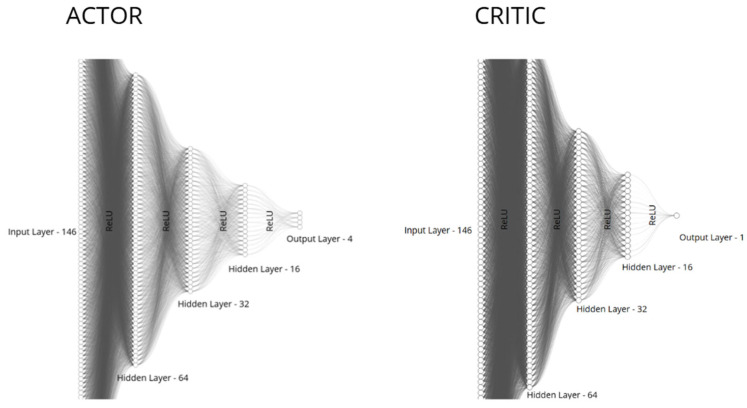
Neural network architecture used in the study for the REINFORCE and PPO configurations.

Figure 4 presents the neural network architecture used in the study. The policy network used in the REINFORCE configuration is a fully connected multilayer perceptron with layer sizes 146, 64, 32, and 16, followed by the continuous action output layer. The output dimension equals to the number of continuous control channels used by the agent, i.e., the action components shown in Figure 3 and mapped to the UAV low-level control inputs. ReLU activation is applied after each hidden layer. In the PPO configuration, the actor uses the same policy-network structure, whereas the critic is implemented as a separate fully connected network with layer sizes 64, 32, and 16 and a scalar output corresponding to the estimated state value.

The use of separate actor and critic networks allows the policy and value function to specialize in different subproblems of learning. The actor is responsible for action generation, whereas the critic estimates expected return and stabilizes updates through advantage computation.

It is important to note that the actor and critic networks need not share the same architecture. They can differ in the number of layers, number of neurons, or other hyperparameters. In Unity ML-Agents, they are typically trained as separate models, and their architecture can be configured independently in the training configuration file. The only element that remains the same across both networks is the ReLU activation function, which is commonly used to introduce nonlinearity, enabling the networks to model complex nonlinear relationships between inputs and outputs and improving their ability to learn expressive feature representations [29,30].

## 3. Results and Discussion

To clarify the evaluation procedure, the experimental study was organized into three consecutive training stages. The purpose of this design was not only to compare REINFORCE and PPO but also to examine how both algorithms behave under increasingly demanding learning conditions. In Stage 1, the agents were trained in a relatively simple setting to establish an initial policy and identify promising hyperparameter configurations. In Stage 2, training continued using the experience gathered in Stage 1 and a more demanding task configuration, enabling assessment of learning stability, adaptation, and convergence under increased complexity. In Stage 3, the selected configurations were further trained in the most difficult scenario, with denser obstacle arrangements and more challenging navigation conditions, to evaluate the robustness of the learned behavior and select the final models for the validation experiment. It should be emphasized that the purpose of this staged experimental design was model selection and comparative assessment of candidate configurations, rather than repeated-seed statistical estimation for a single fixed final setting. Multiple training runs were therefore used to identify the most promising configurations at each stage. In addition, the staged workflow was designed so that the best-performing policy obtained at one difficulty level served as the starting point for the next stage, rather than forcing the agent to learn the full mission complexity from scratch. Although this procedure improves the reliability of configuration selection, it is not equivalent to a formal variability analysis that relies on repeated executions of the same final configuration with different random seeds. This staged procedure was intended to reduce the difficulty of learning in the initial phase and then progressively test whether the algorithms could transfer earlier experience to more complex mission conditions. The comparison between PPO and REINFORCE was designed as a controlled experimental comparison under identical task conditions. Both algorithms were trained in the same virtual environment, with the same mission objective, observation space, reward formulation, operational constraints, and staged-training logic. Therefore, the comparison isolates the effect of the learning algorithm under matched experimental conditions. At the same time, due to stochastic network-weight initialization and stochastic exploration during training, the obtained results should be interpreted as evidence from a controlled algorithmic comparison rather than as a statistically exhaustive repeated-seed benchmark. However, it should be emphasized that the primary goal of this study was not to establish statistically rigorous performance bounds, but to compare algorithmic behavior under identical and controlled mission conditions. Therefore, the presented results should be interpreted as indicative comparative evidence rather than as a statistically complete benchmark. A more rigorous repeated-seed evaluation with confidence intervals and formal significance testing is an important direction for future work.

All experiments were conducted on a fixed hardware platform equipped with an NVIDIA RTX 4070 Ti Super GPU (16 GB VRAM, 8448 CUDA cores, memory bandwidth of 672 GB/s), an Intel i7-14700KF CPU, and 64 GB of system memory. The reported CPU, GPU, and RAM utilization values represent relative resource usage measured on this platform and are provided primarily to compare the computational demands of the evaluated algorithms under identical conditions. These values should not be interpreted as absolute performance indicators, as they depend on the specific hardware configuration used during the experiments.

The reported evaluation measures should be interpreted into two groups. The first group consists of learning-process metrics, which describe how the optimization proceeds during training. These include policy loss, cumulative reward or success-related reward indicator, entropy/exploration, explained variance, episode length, computation time, and hardware-resource usage. The second group consists of mission-level performance metrics that describe the final practical effectiveness of the trained agent, most notably the success rate in the independent validation scenario. This distinction is important because good optimization behavior during training does not automatically imply strong final mission performance.

**Figure 5:**
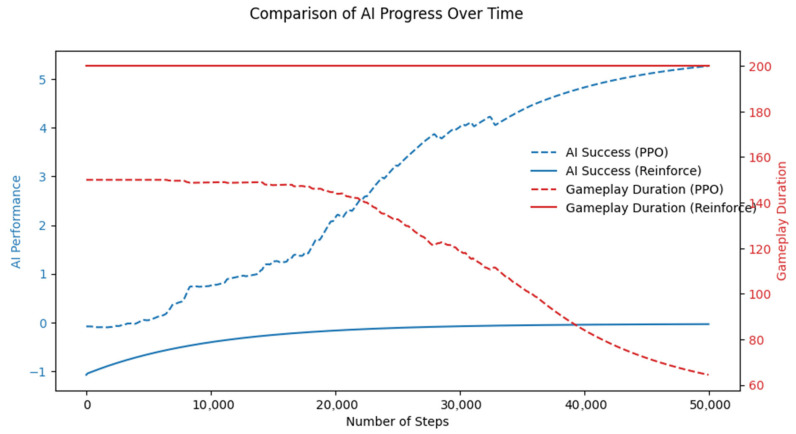
Stage 1: The learning process of both algorithms.

**Figure 6:**
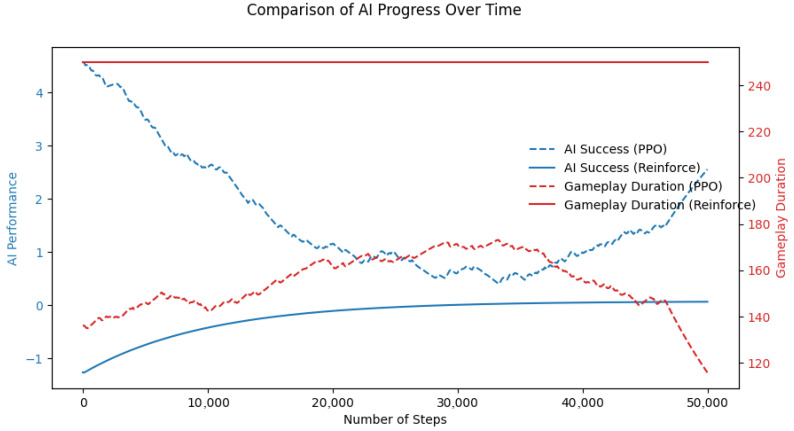
Stage 2 learning process for both algorithms after expanding the continuous action space to include vertical motion (up/down) in addition to forward/backward movement.

**Figure 7:**
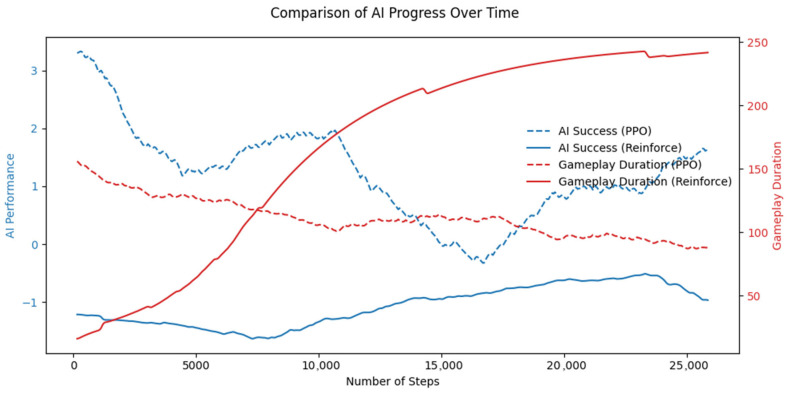
Stage 3 learning process for both algorithms in the presence of environmental obstacles, requiring simultaneous multi-axis continuous control and collision-aware navigation.

In the training plots (Figure 5, Figure 6 and Figure 7), the term “AI Performance” is used as a general indicator of the agent’s learning progress and corresponds to the reward-based performance measure recorded during training, i.e., the cumulative reward (or mean episodic reward, depending on the specific plot). The values shown on the vertical axis therefore reflect the scale of the defined reward function rather than normalized performance scores. As a result, the range of values includes both negative and positive values, which correspond, respectively to penalty-dominated and reward-dominated agent behavior. Therefore, “AI Performance” should be interpreted as an internal training metric reflecting policy quality under the designed reward function, rather than an absolute or directly comparable performance measure.

Policy loss reflects the magnitude and direction of policy updates during optimization. It is useful mainly as an internal learning indicator and should not be interpreted alone as a direct measure of mission quality. Cumulative reward indicates how consistently the agent performs behaviors encouraged by the reward function, such as moving toward the target, avoiding penalties, and maintaining safe flight conditions. Entropy, reported for PPO, quantifies the degree of action randomness and thus the level of exploration: higher entropy indicates more exploratory behavior, whereas lower entropy indicates a more deterministic policy. Explained variance is used only for PPO because PPO employs a critic network; this metric indicates how well the critic predicts returns, with higher values indicating a better fit of the value function. Episode length indicates how many simulation steps are required before the episode terminates; shorter episodes may indicate either faster task completion or faster failure, so this metric must always be interpreted alongside reward and success indicators. CPU, RAM, and GPU utilization, together with computation time, describe the computational cost of training.

The observed advantage of PPO over REINFORCE in the present study can be linked not only to the general properties of the algorithm, but also to the specific requirements of the UAV control task considered here. The mission requires simultaneous continuous control, obstacle avoidance, target-oriented navigation, altitude maintenance, and reaction under partial observability. In such a setting, policy updates based only on Monte Carlo returns, as in REINFORCE, are more sensitive to reward variance and delayed task feedback. By contrast, PPO benefits from the actor–critic structure, which provides a value-based baseline for advantage estimation, and from clipped policy updates, which reduce destructive parameter changes during learning. These mechanisms are especially relevant in the present UAV setup, where the agent must maintain stable multi-axis control while also making sequential navigation decisions under sparse success signals and multiple penalty terms. This task-specific interpretation helps explain why PPO achieved better reward progression, shorter effective episode lengths in the later stages, and higher final mission success under the same observation space, reward structure, and staged-training procedure. In particular, PPO was better suited to this problem because unstable policy updates in a multi-axis UAV-control task can immediately translate into loss of altitude control, collision, or mission failure. By reducing gradient variance through value-based advantage estimation and limiting abrupt policy shifts through clipping, PPO provided a more reliable mechanism for improving control behavior in this safety-constrained and partially observable environment.

The tested hyperparameters also require explicit interpretation. The learning rate determines the magnitude of updates to network parameters: lower values usually improve stability but slow learning, whereas higher values accelerate learning at the risk of instability. The discount factor γ controls the importance of future rewards; higher values encourage long-term planning, whereas lower values emphasize immediate outcomes. In PPO, the number of rollout steps (num_steps) determines how much interaction data is collected before each optimization update, and total_timesteps specifies the overall training budget. The number of minibatches (num_minibatches) determines how the collected data is split during optimization, affecting the granularity of gradient updates. The parameter gae_lambda controls the bias–variance trade-off in Generalized Advantage Estimation: higher values typically provide smoother long-horizon estimates but may increase variance. The clipping coefficient (clip_coef) limits the size of policy updates in PPO and therefore directly affects training stability. The entropy coefficient (ent_coef) controls how strongly exploration is encouraged; larger values promote more exploratory policies. The value-function coefficient (vf_coef) sets the relative importance of critic loss in PPO, and max_grad_norm limits gradient magnitude to prevent unstable updates. In REINFORCE, the parameter std controls the spread of the stochastic action distribution and therefore influences exploration intensity. Finally, max_steps defines the maximum episode length and num_episodes determines the number of episodes used in training.

For this reason, the tables in the following subsections should be read in a structured way. The parameter tables explain the configuration used in each test, while the result tables summarize the observed training behavior under that configuration. Figures showing the training process illustrate the dynamics of reward and episode length over time and are intended to complement the tables by revealing convergence patterns rather than replacing the final mission-level validation.

For methodological consistency, all candidate tests within a given training stage were conducted under the same environment configuration, observation model, action space, reward formulation, and mission constraints. Only algorithm-specific and training hyperparameters were varied between runs. Hyperparameter selection was supported by Optuna-based optimization and complemented by iterative analysis of learning dynamics, convergence stability, and task-level performance. After each stage, the configuration selected for further training was the one that provided the best overall balance between achieved performance and stability under the fixed scenario conditions of that stage.

In each of the four tests listed in Table 2, the agent was allowed to take up to 200 steps per episode and was trained for 250 episodes. The differences between the tests concerned three parameters. The learning_rate, which determines how quickly the network weights are updated. A higher learning_rate means the agent updates its weights more aggressively, so it can learn faster but is also more likely to become unstable or “overshoot” good solutions. A learning_rate rate leads to slower, more cautious learning, which is usually more stable but may require more training steps. The discount factor gamma determines how strongly the agent weights future rewards. A higher gamma (closer to 1) indicates that the agent places greater weight on long-term rewards and plans further into the future. A lower gamma indicates that the agent places greater emphasis on short-term, immediate rewards and is less sensitive to future outcomes. The standard deviation controls the level of randomness in action selection and thus the degree of exploration. A higher standard deviation (Std) makes the policy more stochastic, so the agent explores more and tries a wider range of actions, which can help discover better strategies but also increases variability in performance. A lower standard deviation makes the policy more deterministic, so the agent exploits what it has already learned, explores less, and may become stuck in suboptimal behavior.

Results listed in Table 3 show that test no. 2 has the highest average reward per episode. It can be safely stated that test no. 2, with its parameter settings, is the best among those considered. The only drawback is that it did not manage to reduce the number of steps required to complete an episode and, consequently, the total training time. Resource usage in all test cases for the REINFORCE algorithm is very similar. Test no. 2 is selected for further training.

As shown in Table 4, the experiments used the same rollout length of 150 environment steps per update and a total training budget of 50,000 timesteps across all four tests. The learning_rate varies significantly between the tests: Test 1 uses a very small learning_rate, leading to slow and conservative updates, while Test 4 applies the highest learning_rate, enabling faster but more aggressive learning. The discount gamma factor γ ranges from 0.891 to 0.985, with Test 2 placing greater emphasis on long-term rewards, whereas Test 1 focuses more on immediate rewards. The number of minibatches, num_minibatches is fixed at 5 in all experiments, ensuring that the collected data is split in the same way during optimization. The GAE parameter λ varies slightly across tests, controlling the bias–variance trade-off in advantage estimation, where higher values reduce bias but increase variance. The clipping coefficient clip_coef remains relatively small across all tests, thereby helping to maintain stable policy updates. Entropy (ent_coef) is lower in Tests 2 and 3, resulting in more deterministic policies, whereas it is higher in Test 4, encouraging greater exploration. The value-function loss coefficient vf_coef is higher in Tests 1 and 4, indicating a stronger focus on accurate value estimation, whereas tests 2 and 3 maintain a more balanced emphasis between policy and value learning. The max_gradient_norm further controls training stability by limiting the magnitude of gradients, with lower values enforcing stricter clipping and higher values allowing larger updates.

As we can see in Table 5, Test 4, which uses the highest learning_rate, also features a slightly lower discount factor than Test 2 but a higher GAE lambda, placing greater emphasis on long-term reward estimation. Entropy remains high and continues to increase during training, and the value-function coefficient vf_coef is the largest among all experiments, meaning that the critic strongly influences the learning process. In addition, a large max_grad_norm allows for larger parameter updates. As a result, Test 4 achieves the highest cumulative reward and the highest explained variance, indicating strong critic performance. However, despite reaching high rewards in the shortest time with stable resource usage, Test 2 is selected for further training due to its greater overall stability and higher training efficiency.

As shown in Table 6, the two algorithms exhibit similar policy losses, so their updates are of comparable magnitude. However, the PPO algorithm achieved nearly twice the reward and required substantially less time to achieve this performance. The main difference is that the REINFORCE algorithm uses a cumulative reward over 200 steps and does not use an additional critic to stabilize learning. As a result, its hardware usage is significantly higher. PPO performs significantly better in terms of effectiveness, hardware requirements, and time efficiency. From Figure 6, it is evident that after approximately 25,000 steps, PPO exhibits a clear trade-off between reward and episode length, whereas with REINFORCE, the episode length remains constant and the reward improves only slightly.

For Stage 2 of the REINFORCE algorithm, as shown in Table 7, the agent was allowed to take up to 250 steps per episode and was trained for 260 episodes per test. As in Stage 1, the tests differed in three key hyperparameters: the learning_rate, the discount factor gamma, and the standard deviation Std.

The results in Table 8 show that, despite differences in hyperparameters across tests, the average episode length remained approximately constant at approximately 250 steps across all four configurations. This indicates that the agent did not learn to complete episodes more efficiently and consistently used the maximum allowed number of steps. At the same time, the success rates are negative across all tests, indicating that the agent’s overall performance with respect to the adopted reward metric deteriorated or remained unsatisfactory in this training stage. The exploration level closely tracks the selected standard deviation values in Table 7, confirming that this parameter effectively controls the policy’s stochasticity. Test 4 exhibits the lowest exploration and the highest learning_rate. The reward does not increase dynamically due to the reduced exploration. Unfortunately, this cannot be considered an advantage in this case, because the cumulative reward is very low, and a stable but poor policy is not an optimal solution. Therefore, Test 2 is selected for further training.

Table 9 shows that the learning_rate values vary significantly: Test 2 uses the lowest value, which favors stable, conservative updates, whereas Test 4 uses the highest value, which accelerates learning but increases the risk of instability. Tests 1 and 3 occupy intermediate positions. The discount factor gamma is highest in Tests 1 and 2, which increases the emphasis on long-term rewards, whereas in Test 3 it is the lowest, causing the agent to focus more on short-term rewards. The gae_lambda parameter is close to 1 across all tests, with the highest value in Test 3, which extends the advantage estimation horizon and increases variance. All configurations use relatively small clip_coef values, with the smallest in Test 4, which strongly constrains policy updates. The entropy coefficient ent_coef is lowest in Test 2 (more deterministic policy), highest in Tests 3 and 4 (strong exploration), and moderate in Test 1. The vf_coef values are highest in Tests 1 and 3, indicating a stronger focus on accurate value-function fitting, whereas in Tests 2 and 4, they are lower, balancing value and policy learning. The max_grad_norm parameter is highest in Test 1, allowing larger parameter updates, and lowest in Test 4, resulting in more constrained parameter updates per optimization step.

Table 10 shows that Test 2 achieves the best trade-off between policy quality and training stability: it has the highest explained variance, a relatively high cumulative reward, and shorter training time than Test 1, while maintaining a more deterministic policy. Test 1 yields decent results but is slower and less efficient. Test 3 performs the worst—despite high entropy, it achieves the lowest reward and the lowest explained variance, indicating poor critic fit. Test 4 is the most unstable: an aggressive learning_rate combined with a very small clip_coef and high entropy leads to large policy instability, negative explained variance, and short, unsuccessful episodes, despite a moderate average reward. Test no. 2 is selected for further training due to its best achieved results and its stability, which is key for continued learning.

Both algorithms exhibit very similar policy losses, as shown in Table 11. However, the PPO algorithm performs better, which was expected in such a complex environment. PPO uses more resources but has a much shorter training time. Its efficiency is high, and episode duration decreases over time, unlike REINFORCE. In the case of PPO in Figure 6, the AI’s success steadily improves during training, while the episode duration gradually decreases. This indicates that the agent learns to solve the task more efficiently, achieving its goals in fewer steps as training progresses. In contrast, the REINFORCE algorithm exhibits much slower improvement in AI performance, and episode duration remains nearly constant throughout training, suggesting limited learning progress.

Looking at Table 12 and Table 13 the following conclusions can be drawn. The policy loss for both tests is very similar in the initial phase of training. However, the policy in Test 2 converges to zero more quickly and then stabilizes, whereas in Test 1 it remains at high values with frequent increases, indicating large fluctuations in the policy. Test 2 yields higher rewards and greater efficiency than Test 1. This may be because Test 2 involves less exploration and relies more on prior experience. Test 1 is characterized by high variability in decisions and by episodes that often terminate quickly with failure. As a result, it achieved the shortest training time so far. Nevertheless, Test 2 achieved better results across all categories and is therefore selected as the final trained model for the REINFORCE algorithm.

In both cases, Table 14 and Table 15 indicate that rewards are lower and more variable due to numerous obstacles. In such situations, the agent may sometimes have to take a longer route or identify a gap to reach the goal. In the early training phases, the models explore different obstacle-avoidance strategies. Test 2 substantially outperforms Test 1 in predicting the Critic’s estimate. The random placement and high number of obstacles hinder the model in this task, so it continues to predict values with uncertainty. Test 2 has significant difficulty with this. A possible cause is an excessively high vf_coef value, set close to the upper limit. In both tests, the models eventually stabilized toward the end of training but exhibited considerable fluctuations during training, still attempting to respond in various ways to changing conditions. Test 2 had a shorter training period but achieved lower rewards for most of the training. Test 1 is therefore selected as the final PPO model.

According to Table 16, the PPO algorithm makes decisions more quickly and produces less chaotic actions. The REINFORCE algorithm, despite lacking additional mechanisms, performs relatively well in such a complex environment, which is a positive surprise. It consumes fewer resources, but the rewards are still lower than in the case of PPO, and the episode duration increases throughout training. PPO, as shown in Figure 7, eventually “bounces back” and plays the game more efficiently. One could consider extending the training time in the final learning phase, at the cost of reduced generalization. To further assess the final mission-level effectiveness of both approaches, selected Stage 3 policies of each algorithm were evaluated in a previously unseen mission scenario over 100 mission attempts.

Selected Stage 3 policies of each algorithm were evaluated in a previously unseen mission scenario over 100 mission attempts. In this scenario, the UAV was required to maintain a prescribed flight altitude, detect a designated ground target location, and perform the target-approach task while avoiding obstacles such as trees. The results summarized in Table 17 show that, within the considered validation scenario, the PPO configuration with an Actor–Critic architecture outperformed REINFORCE in terms of mission success, flight stability, collision avoidance, and time-to-target. The large difference in success rates suggests that PPO provided a more robust control policy under the operating conditions considered in this study.

## 4. Future Work

The concept warrants further development by increasing scenario complexity, refining the reward function, and exploring transfer to real-world UAV platforms. Future work should include broader validation in multiple unseen environments, robustness analysis under varying initial conditions, and structured sensitivity studies for the observation-space design and reward-weight selection. In particular, a formal ablation analysis should be conducted to determine which observation components are essential, which are redundant, and how the dimensionality and composition of the observation vector influence convergence, robustness, and final mission performance. An important extension would also be to validate the simulation environment against real-world flight behavior or reference dynamic models to assess how accurately the adopted physics-informed setup reflects realistic UAV operation. Another promising direction is to extend the framework to cooperative multi-UAV missions and incorporate additional environmental factors, such as wind, rain, and degraded visibility. The staged training procedure presented in this work may also be extended toward more diverse mission objectives, more complex obstacle layouts, and adaptive scenario generation. The system may further serve as a platform for research, training, and education on AI-based decision-making in a controlled, game-like environment.

With the growing use of unmanned systems and artificial intelligence in safety-critical and infrastructure-related domains, the proposed approach is particularly relevant. Methods for autonomous, safe, and efficient UAV control developed and validated in simulation may support the development of advanced decision-support tools and autonomous functions for applications such as infrastructure inspection, monitoring, search-and-rescue operations, and environmental assessment.

## 5. Conclusions

This work presented a complete reinforcement learning-based decision system for autonomous control of a multirotor UAV in a three-dimensional simulation environment, designed specifically for operation under degraded navigation (GPS-denied) and limited communication. Instead of relying on classical, pre-programmed trajectories and control rules, the drone learned a control policy through interaction with its environment, receiving feedback in the form of rewards and penalties. This enabled the gradual acquisition of stable flight, obstacle-avoidance, and mission-execution skills under uncertainty and partial observability.

Compared with many prior studies, the main contribution of this work lies not in proposing a new learning algorithm but in integrating a mission-oriented UAV control problem, a realistic Unity/ML-Agents simulation setup, a tailored observation and reward design, and a staged training-and-selection workflow into a single coherent experimental framework. Second, the work provided a systematic comparison of two policy-gradient configurations: classical REINFORCE with a single actor network and PPO with an Actor–Critic architecture. In the PPO setup, Generalized Advantage Estimation and policy update clipping were employed, significantly improving training stability and data efficiency. A dedicated neural network architecture (separate actor and critic models) was implemented and tailored to UAV sensing and continuous control, rather than relying on generic default models.

An important contribution was also the multi-stage training strategy, in which task complexity was gradually increased: from simple navigation scenarios through environments with obstacles to final missions requiring precise approach to a ground target location in a previously unknown environment. This curriculum-style progression reduced the complexity of training samples and stabilized learning, fostering the emergence of complex behaviors. In addition, a staged model-selection procedure was used, in which multiple policy instances with different hyperparameters and initializations were trained and compared across successive difficulty levels, and the best-performing policy was selected for further refinement.

Although the final validation was conducted in a single previously unseen environment configuration with a fixed obstacle layout and target location, the UAV started each mission from different initial positions. As a result, the trained policy had to generate different approach trajectories and decision sequences across attempts. Therefore, the reported results provided evidence of robustness to varying initial conditions within an unseen scenario, rather than comprehensive generalization across multiple randomized environments. These results suggest that properly designed DRL-based decision systems may provide a viable alternative to classical controllers in complex and uncertain operating conditions, particularly when GPS and communication links are limited or denied.

The presented solution had both scientific and practical significance. From a research perspective, it provided a coherent case study of applying policy-gradient methods (REINFORCE vs. PPO) in a realistic UAV scenario, integrating a continuous action space, an advanced reward function, GAE, and curriculum training. On the practical side, the proposed architecture and training methodology represented a step toward fully autonomous, disruption-resilient UAV systems applicable to civilian and safety-critical environments in which traditional GPS- and operator-dependent navigation systems may fail.

## Figures and Tables

**Table 2:** Stage 1: Presentation of parameter values for individual tests of the REINFORCE algorithm.

No.	Parameter Name	Test 1	Test 2	Test 3	Test 4
1.	Max_steps	200	200	200	200
2.	Num_episodes	250	250	250	250
3.	Learning_rate	0.00096	0.00087	0.00060	0.00091
4.	Gamma	0.975	0.923	0.950	0.857
5.	Std	0.136	0.290	0.918	0.510

REINFORCE: Stage 1.

**Table 3:** Stage 1: Results of the mean values for the presented tests of the REINFORCE algorithm.

No.	Parameter Name	Test 1	Test 2	Test 3	Test 4
1.	Policy loss	−0.1218	0.0333	0.1281	0.0126
2.	Success rate	0.7452	1.5712	0.6092	0.2491
3.	Exploration	0.1351	0.2881	0.9103	0.5090
4.	Episode length (steps)	200	200	200	200
5.	CPU utilization (%)	7.4264	7.4980	7.4916	7.6528
6.	Memory usage (%)	24.1736	24.2008	24.2456	24.2712
7.	GPU utilization (%)	44.50	45.06	44.99	44.80
8.	Compute time (min:s)	17:00	17:00	17:00	17:00

**Table 4:** Stage 1: Presentation of parameter values for individual tests of the PPO algorithm. PPO: Stage 1.

No.	Parameter Name	Test 1	Test 2	Test 3	Test 4
1.	Num_steps	150	150	150	150
2.	Total_timesteps	50,000	50,000	50,000	50,000
3.	Learning_rate	0.000058	0.00021	0.00024	0.00079
4.	gamma	0.891	0.985	0.934	0.956
5.	Num_minibatches	5	5	5	5
6.	Gae_lambda	0.857	0.805	0.800	0.829
7.	Clip_coef	0.278	0.227	0.233	0.217
8.	Ent_coef	0.051	0.011	0.011	0.067
9.	Vf_coef	0.902	0.498	0.568	0.996
10.	Max_grad_norm	0.758	0.227	0.143	0.466

**Table 5:** Stage 1: Results of the mean values for the presented tests of the PPO algorithm.

No.	Parameter Name	Test 1	Test 2	Test 3	Test 4
1.	Policy loss	−0.1186	0.0354	−0.0257	−0.1723
2.	Cumulative reward	2.2850	3.8658	2.8634	4.0744
3.	Entropy (Exploration)	2.8926	2.7481	2.7903	3.8748
4.	Explained variance	0.1174	0.7281	0.4558	0.7358
5.	Episode length (steps)	141.68	110.20	129.66	83.18
6.	CPU utilization (%)	8.6125	9.7720	7.5850	7.3142
7.	RAM usage (%)	23.5098	23.9224	24.1446	24.6076
8.	GPU utilization (%)	36.13	33.74	35.91	34.39
9.	Computation time (min:s)	17:30	12:18	16:00	10:31

**Table 6:** Stage 1: Comparison of selected algorithm tests.

No.	Parameter Name	REINFORCE	PPO
1.	Policy loss	0.0333	0.0354
2.	Cumulative reward	1.5712	3.8658
5.	Episode length (steps)	200	110.20
6.	CPU utilization (%)	7.4980	9.7720
7.	RAM usage (%)	24.2008	23.9224
8.	GPU utilization (%)	45.06	33.74
9.	Computation time (min:s)	17:00	12:18

**Table 7:** Stage 2: Presentation of parameter values for individual tests of the REINFORCE algorithm.

No.	Parameter Name	Test 1	Test 2	Test 3	Test 4
1.	Max_steps	250	250	250	250
2.	Num_episodes	260	260	260	260
3.	Learning_rate	0.00017	0.00059	0.00031	0.00044
4.	Gamma	0.827	0.945	0.968	0.860
5.	Std	0.480	0.371	0.478	0.286

REINFORCE: Stage 2.

**Table 8:** Stage 2: Results of the mean values for the presented tests of the REINFORCE algorithm with the experience gathered in Stage 1 and current stage.

No.	Parameter Name	Test 1	Test 2	Test 3	Test 4
1.	Policy loss	−0.0743	−0.0521	−0.1743	−0.0205
2.	Success rate	−1.5367	−0.5285	−0.6767	−1.3856
3.	Exploration	0.4783	0.3681	0.4775	0.2852
4.	Episode length (steps)	250.00	250.00	249.78	250.00
5.	CPU utilization (%)	8.6430	8.4830	8.2828	8.9610
6.	Memory usage (%)	24.3185	24.6085	25.0480	25.3430
7.	GPU utilization (%)	38.38	37.32	35.38	31.32
8.	Compute time (min:s)	23:00	23:00	22:55	23:00

**Table 9:** Stage 2: Presentation of parameter values for individual tests of the PPO algorithm.

No.	Parameter Name	Test 1	Test 2	Test 3	Test 4
1.	Num_steps	200	200	200	200
2.	Total_timesteps	65,000	65,000	65,000	65,000
3.	Learning_rate	0.000124	0.00003534	0.000438	0.000714
4.	gamma	0.986	0.984	0.893	0.942
5.	Num_minibatches	5	5	5	5
6.	Gae_lambda	0.872	0.829	0.977	0.913
7.	Clip_coef	0.224	0.217	0.206	0.161
8.	Ent_coef	0.042	0.018	0.075	0.040
9.	Vf_coef	0.732	0.444	0.735	0.526
10.	Max_grad_norm	0.712	0.652	0.580	0.546

PPO: Stage 2.

**Table 10:** Stage 2: Results of the mean values for the presented tests of the PPO algorithm with the experience gathered in Stage 1 and current stage.

No.	Parameter Name	Test 1	Test 2	Test 3	Test 4
1.	Policy loss	−0.162581	−0.045691	−0.277624	−0.658605
2.	Cumulative reward	1.1462	1.2742	0.1811	0.7179
3.	Entropy (Exploration)	4.8706	4.9381	8.8204	10.3590
4.	Explained variance	0.4158	0.5585	0.0679	−3.1234
5.	Episode length (steps)	162.88	143.78	120.29	105.50
6.	CPU utilization (%)	8.114	10.168	8.980	9.412
7.	RAM usage (%)	27.225	29.003	23.945	24.068
8.	GPU utilization (%)	36.55	35.79	35.87	36.82
9.	Computation time (min:s)	22:32	18:23	16:27	14:16

**Table 11:** Stage 2: Comparison of selected algorithm tests.

**No.**	**Parameter Name**	**REINFORCE**	**PPO**
1.	Policy loss	−0.0521	−0.045691
2.	Cumulative reward	−0.5285	1.2742
5.	Episode length (steps)	250.00	143.78
6.	CPU utilization (%)	8.4830	10.168
7.	RAM usage (%)	24.6085	29.003
8.	GPU utilization (%)	37.32	35.79
9.	Computation time (min:s)	23:00	18:23

**Table 12:** Stage 3: Presentation of parameter values for individual tests of the REINFORCE algorithm.

No.	Parameter Name	Test 1	Test 2
1.	Max_steps	250	250
2.	Num_episodes	260	260
3.	Learning_rate	0.000737	0.000918
4.	Gamma	0.876	0.889
5.	Std	0.110	0.101

REINFORCE: Stage 3.

**Table 13:** Stage 3: Results of the mean values for the presented tests of the REINFORCE algorithm with the experience gathered in Stage 1, 2 and the current stage.

No.	Parameter Name	Test 1	Test 2
1.	Policy loss	0.4968	0.1630
2.	Success rate	−1.187	−1.018
3.	Exploration	0.1104	0.1012
4.	Episode length (steps)	68.08	210.93
5.	CPU utilization (%)	7.842	8.269
6.	Memory usage (%)	22.363	22.732
7.	GPU utilization (%)	36.79	24.93
8.	Compute time (min:s)	4:34	14:07

**Table 14:** Stage 3: Presentation of parameter values for individual tests of the PPO algorithm.

No.	Parameter Name	Test 1	Test 2
1.	Num_steps	200	200
2.	Total_timesteps	65,000	65,000
3.	Learning_rate	0.000576	0.000417
4.	gamma	0.948	0.857
5.	Num_minibatches	5	5
6.	Gae_lambda	0.843	0.862
7.	Clip_coef	0.127	0.211
8.	Ent_coef	0.0125	0.0130
9.	Vf_coef	0.499	0.971
10.	Max_grad_norm	0.641	0.421

PPO: Stage 3.

**Table 15:** Stage 3: Results of the mean values for the presented tests with the experience gathered in Stage 1, 2 and the current stage.

No.	Parameter Name	Test 1	Test 2
1.	Policy loss	−0.0200	−0.04432
2.	Cumulative reward	1.1669	0.5586
3.	Entropy (Exploration)	9.2299	11.7241
4.	Explained variance	0.2925	−0.2089
5.	Episode length (steps)	79.55	73.26
6.	CPU utilization (%)	10.39	11.05
7.	RAM usage (%)	19.54	29.90
8.	GPU utilization (%)	35.39	35.57
9.	Computation time (min:s)	11:25	7:57

**Table 16:** Stage 3: Comparison of selected algorithm tests.

No.	Parameter Name	REINFORCE	PPO
1.	Policy loss	0.1630	−0.0200
2.	Cumulative reward	−1.018	1.1669
5.	Episode length (steps)	210.93	79.55
6.	CPU utilization (%)	8.269	10.39
7.	RAM usage (%)	22.732	19.54
8.	GPU utilization (%)	24.93	35.39
9.	Computation time (min:s)	14:07	11:25

**Table 17:** Comparison of the effectiveness of the trained models using each algorithm.

No.	Algorithm	Successes	Failures	Success Rate
1.	REINFORCE	10	90	10%
2.	PPO	71	29	71%

## Data Availability

The authors confirm that the data supporting the findings of this study are available within the article.
